# Determination of Organophosphate Ester Metabolites in Seafood Species by QuEChERS-SPE Followed by LC-HRMS

**DOI:** 10.3390/molecules27238635

**Published:** 2022-12-06

**Authors:** Míriam Hidalgo-Serrano, Francesc Borrull, Eva Pocurull, Rosa Maria Marcé

**Affiliations:** Department of Analytical Chemistry and Organic Chemistry, Universitat Rovira i Virgili, Marcel·lí Domingo s/n, 43007 Tarragona, Spain

**Keywords:** seafood, organophosphate diesters, QuEChERS, liquid chromatography, high-resolution mass spectrometry, solid-phase extraction

## Abstract

Organophosphate triesters are compounds widely used in industries and are ubiquitous in the environment, where they can be transformed into organophosphate diesters. Some organophosphate diesters are also used by industry. Several studies suggest organophosphate diesters can have toxic effects for reproduction, and hazardous and mutagenic properties. Due to the impact these compounds can have on marine biota and human beings through the consumption of fish and shellfish, it is necessary to study their presence in widely consumed seafood species. We therefore developed an analytical method for determining six of the most common organophosphate diesters in seafood. The procedure is based on the Quick, Easy, Cheap, Effective, Rugged and Safe extraction method and a solid phase extraction clean-up, followed by liquid chromatography coupled to high-resolution mass spectrometry. The method was optimised and validated for seafood with different lipid content, providing satisfactory relative recoveries (from 89 to 138%) and limits of detection (1.0–50 ng g^−1^ dry weight), as well as repeatability values (RSD% (*n = 5*, 100 ng g^−1^ (dry weight)) lower than 15%. Eight seafood species were analysed using this method and two organophosphate diesters were detected and quantified in all the samples, demonstrating the suitability of the method.

## 1. Introduction

Organophosphate (OP) triesters are high-production volume chemicals mainly used as flame retardant additives and plasticisers in consumer products such as upholstered furniture, textiles, electronic devices and plastics, among others [1]. Over the past few decades, the production of these chemicals has increased considerably after the use of brominated flame retardants was banned due to their persistence, toxicity and bioaccumulation potential [2]. OP triesters are usually applied to the products by physical mixing, and are not chemically bonded; therefore, they easily leach into the environment during the lifetime of the products. As a result, OP triesters are ubiquitous and have been reported in a wide variety of matrices, such as indoor dust [3,4], sediments [5,6], surface waters [2,7,8] and biota [2,9]. This could be a cause for concern, considering that some of them are hazardous and suspected endocrine disruptors [2,10].

Several in vivo [11,12] and in vitro [13,14] studies report that OP triesters are rapidly hydrolysed by living organisms. OP diesters are some of the most commonly detected metabolites and could be used as biomarkers of exposure to these contaminants. Moreover, some OP diesters, such as dibutyl phosphate (DNBP), bis(2-ethylhexyl) phosphate (BEHP) and diphenyl phosphate (DPHP), are also used as chemical additives in several industrial processes. Therefore, industrial waste is another source of environmental contamination for these compounds [15].

Similar to OP triesters, some OP diesters have shown the potential to cause developmental cardiac defects [16], circulatory failures [17], and general teratogenicity [18]. Some even demonstrate a stronger ability to alter gene expression than their parent products [19] and can have hazardous effects on the endocrine system [20]. For this reason, the European Chemicals Agency (ECHA) consider bis(2-chloroethyl) phosphate (BCEP), bis(2-butoxyethyl) phosphate (BBOEP) and DPHP as substances that could be of concern for the industry. ECHA indicates that these compounds are suspected to be hazardous and persistent in aquatic environments as well as mutagens and toxic for reproduction [21].

More specifically, marine environments are very delicate and sensitive to urban, agricultural and industrial sources of contamination. Taking into account that plastic and microplastic contamination is a growing problem worldwide [22], particular attention should be paid to the effect that organic contaminants released from these materials (such as OP esters) could have on marine organisms. However, there is little information on determining these compounds or the occurrence of OP diesters in living organisms. This information should therefore be extended to seafood as it is a very common dietary exposure route to these contaminants for humans [23].

Only a few studies report analytical methods for determining OP diesters in fish. The target compounds are usually extracted by ultrasound extraction [24,25]; however, pressurised liquid extraction has also been used [26]. The extraction procedures are frequently followed by solid phase extraction (SPE) clean-up with weak anion exchange cartridges [24,26]. Then, the extracts are analysed by liquid chromatography (LC), usually coupled to tandem mass spectrometry (MS/MS) because they need to be derivatised to be analysed by gas chromatography [27].

With the aim of filling the information gap regarding the presence of OP diesters in widely consumed seafood species, we developed a simple method for determining six OP diesters using the Quick, Easy, Cheap, Effective, Rugged and Safe (QuEChERS) extraction method followed by LC coupled to high-resolution mass spectrometry (LC-HRMS). Moreover, we tested several strategies for cleaning up the extracts, which usually have high matrix effects (ME) due to the complexity of the seafood matrices.

## 2. Materials and Methods

### 2.1. Reagents and Standards

The solid standards of bis(2-chloroethyl) phosphate (BCEP), diphenyl phosphate (DPHP), dibutyl phosphate (DNBP), bis(1,3-dichloropropyl) phosphate (BDCIPP), bis(2-butoxyethyl) phosphate (BBOEP), bis(2-ethylhexyl) phosphate (BEHP) and the surrogates d_8_-BCEP, d_10_-DPHP, d_18_-DNBP, d_10_-BDCIPP and d_8_-BBOEP were purchased from LGC (Teddington, Middlesex, UK). Stock solutions of the individual standards were prepared in methanol (MeOH) and stored at −23 °C.

Acetonitrile (ACN), acetone and MeOH of HPLC grade were provided by J.T. Baker (Deventer, the Netherlands). Hydrochloric acid (HCl), water and MeOH of MS grade for the mobile phase were purchased from Scharlab (Barcelona, Spain). Ultrapure water was obtained with an ultrapure water purification system (Merck Millipore, Darmstadt, Germany). Acetic acid and ammonium acetate were purchased from Sigma-Aldrich (Saint Louis, Missouri, USA). The extraction salt packets for the original QuEChERS (QUEXTORAK1), EN (QUEXTENAK1) and AOAC (QUEXTAOAK1) methods were supplied by Scharlab.

### 2.2. Sampling

Eight seafood species were obtained from several local fish markets in Tarragona, Spain: *Scomber scombrus* (mackerel), *Merluccius merluccius* (hake), *Solea solea* (sole), *Gadus morhua* (cod), *Loligo Vulgaris* (squid), *Sardina pilchardus* (sardine), *Thunnus thynnus* (tuna) and *Salmo salar* (salmon). All samples were cleaned, filleted and frozen. A Genevac miVac Duo sample concentrator with a SpeedTrap freeze-drying system (Ipswich, UK) was used to lyophilise the samples, which were then homogenised and sieved (500 µm).

### 2.3. QuEChERS Extraction and Clean-Up

A total of 100 mg of freeze-dried sample spiked with 400 ng of surrogate standards was weighted in 50 mL polypropylene tubes (ThermoFisher Scientific, Waltham, MA, USA) and 10 mL of ultrapure water was added to the tube. The mixture was vortexed for 1 min and 10 mL of acetone were added before the tube was vortexed again. Then, an extraction salt packet for the original QuEChERS method (Scharlab), containing 4 g of anhydrous magnesium sulphate and 1 g of sodium chloride, was added to the mixture. The tube was shaken by hand, vortexed again for 5 min and centrifuged at 7000 rpm for 5 min (Hettich Universal 32R, Tuttlingen, Germany). The extraction was repeated once more and the resulting extracts were combined.

The mixture was concentrated to ~5 mL with a miVac Duo sample concentrator. Then, 10 mL of ultrapure water were added and the mixture was vortexed to carry out an SPE clean-up step. Briefly, homemade 2 g PSA cartridges were conditioned with 5 mL MeOH followed by 5 mL ultrapure water/acetone 2:1 (*v*/*v*). The ~15 mL extracts were then loaded onto the cartridges and the analytes were eluted with 5 mL of MeOH 5% NH_4_OH. Finally, the extracts were concentrated to ~200 µL with the miVac Duo sample concentrator, taken up to 1 mL with ultrapure water and injected into the LC-HRMS instrument.

### 2.4. Liquid Chromatography Coupled to High-Resolution Mass Spectrometry

An Accela 1250 UHPLC system from Thermo Scientific (Bremen, Germany) equipped with an Accela Autosampler and a quaternary pump was used for the chromatographic analyses. The system was coupled to an ExactiveOrbitrapTM mass spectrometer (Thermo Scientific) equipped with a heated electrospray ionisation source (HESI) and an HCD collision cell. The chromatographic separation was carried out with an Ascentis Express C_18_ fused-core column (100 mm × 2.1 mm i.d., 2.7 µm particle size) from Sigma-Aldrich. The initial mobile phase was 92% solvent A (2 mM ammonium acetate in water) and 8% solvent B (MeOH), with a flow rate of 200 µL min^−1^, which was held constant for 4.5 min. The gradient was then increased to 90% of B in 9 min and to 100% of B in 0.5 min. It remained constant for 5 min before returning to the initial conditions. The injection volume was 20 µL, and the sample tray and column oven temperatures were 10 °C and 30 °C, respectively.

Mass spectra were acquired in negative mode and the source parameters were optimised to obtain the highest response for all target compounds. The spray voltage was set at −2.5 kV, capillary voltage at −20 V, tube lens voltage at −70 V, and skimmer voltage at −20 V. The sheath and auxiliary gas flow rates were set at 40 and 5 A.U., respectively, while the heater temperature was set at 425 °C and the capillary temperature at 250 °C. For HRMS measurements, two scan events took place in a single time window, a full scan at 50,000 FMHW with 250 ms of injection time, and a fragmentation scan at 10,000 FWHM with 50 ms of injection time using a collision voltage of 25 eV in the HCD cell.

## 3. Results and Discussion

### 3.1. Liquid Chromatography Coupled to High-Resolution Mass Spectrometry

To optimise the HRMS conditions, the gas flow rates, voltages and temperature parameters were tested to obtain the highest response for all compounds. To do so, 1 mg L^−1^ of individual standard solutions were directly infused into the system with a 1:1 (*v*/*v*) composition of water and MeOH. For all compounds, the highest response was obtained in negative ionisation mode for [M − H]^−^ ion. Similarly, the optimum fragmentation energy was selected to obtain the highest response for all quantifier and qualifier ions, which are mainly in accordance with the literature except for those of BCEP and BDCIPP [25,26,28]. The product ions selected for these two compounds when working with MS/MS are usually *m*/*z* 35 and 37. However, the Exactive high-resolution mass spectrometer only allows *m*/*z* values higher than 50; hence, we selected just one qualifier ion for these compounds. The ions selected for quantification and confirmation purposes can be seen in Table 1.

Even though the chromatographic separation of organophosphate diesters can be carried out with HILIC [5] or biphenyl [29] columns, C_18_ is the most commonly used stationary phase [15,25,26]. Hence, we chose an Ascentis Express C_18_ fused-core column (100 mm × 2.1 mm i.d., 2.7 µm particle size) to separate the target compounds. Moreover, OP diesters have usually been separated in reversed-phase columns either at acidic pH [30,31] or with ammonium acetate [3,9,29]. In this study, we compared three different mobile phases to determine the optimal conditions for detecting and separating OP diesters: 0.1% formic acid in water, 0.1% acetic acid in water and 2 mM ammonium acetate in water. At first, we carried out the separation with a very simple gradient. The initial mobile phase was 92% solvent A (one of the three tested aqueous phases) and 8% solvent B (MeOH) with a flow rate of 200 µL min^−1^, which was held constant for 5 min. The gradient was then increased to 100% of B in 15 min and it remained constant for 5 min before returning to the initial conditions.

**Figure 1:**
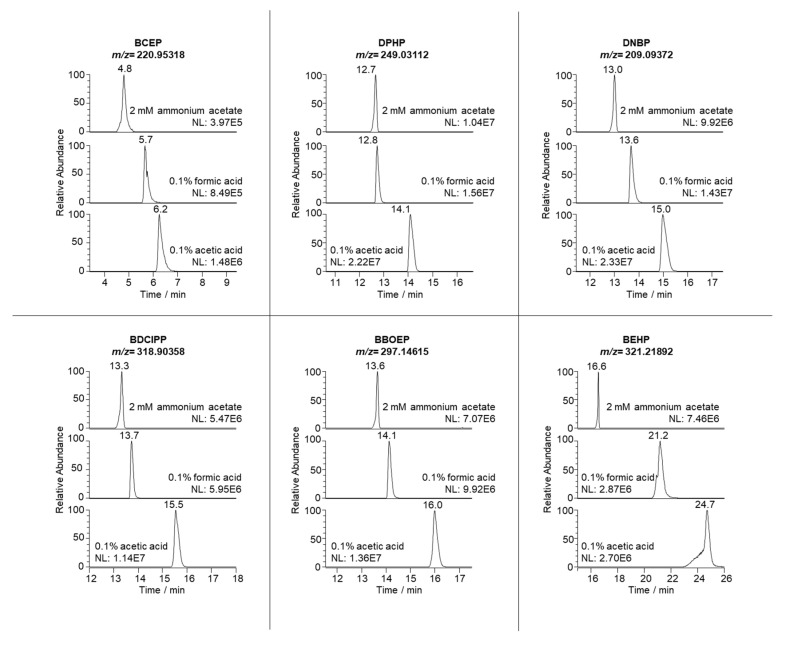
LC-HRMS extracted ion chromatograms of the injection of a standard mixture solution (1 mg L^−1^), using three different additives in the mobile phase.

As can be seen in Figure 1, the chromatogram using acetic acid as a modifier for solvent A resulted in overall broad peaks, especially for BEHP, which showed pronounced peak fronting. The use of formic acid reduced that peak fronting but led to peak tailing, especially for BCEP. Using 2 mM ammonium acetate in water as a mobile phase (pH ~7) greatly reduced peak broadening and, even though it caused slight peak fronting, it yielded a better peak resolution. Therefore, we selected this aqueous phase as optimal for separating the OP diesters. We also modified the gradient elution to reduce the analysis time (finally choosing the gradient described in Section 2.4) and confirmed that the selected HRMS conditions still yielded the highest response possible for all compounds.

Then, instrumental quality parameters and standard calibration curves were calculated for each of the studied compounds taking into account the response of the quantifier ion. Instrumental limits of detection (ILOD) were determined as the concentration at which the response of the quantifier ion was three times the signal-to-noise ratio. The instrumental limits of quantification (ILOQ) corresponded to the lowest concentration of the standard calibration curve. Linear ranges were established for each compound between ILOQ and 1000 µg L^−1^. The ILOD and ILOQ values ranged from 0.5 to 2.5 µg L^−1^ and from 1.0 to 5.0 µg L^−1^, respectively, for all compounds except for BCEP, for which ILOD was 7.5 µg L^−1^ and ILOQ was 10 µg L^−1^.

### 3.2. QuEChERS Extraction

Extraction kits for the original QuEChERS method (containing 1 g sodium chloride and 4 g anhydrous magnesium sulphate), as well as the official EN method (containing 1 g sodium chloride, 1 g sodium citrate, 0.5 g sodium hydrogen citrate sesquihydrate and 4 g anhydrous magnesium sulphate) and the AOAC method (containing 1.5 g sodium acetate and 6 g anhydrous magnesium sulphate) were tested to achieve the most efficient extraction of the target compounds. Extraction recoveries (R_ext_) were evaluated to select the best extraction method by comparing the response of the compounds in the final extract for samples spiked at 1000 ng g^−1^ dry weight (d.w.) before the extraction, with the response obtained for extracts spiked at the same concentration after that step. To do so, 1 g of lyophilised sample was hydrated with 10 mL of ultrapure water and the mixture was vortexed for 1 min. Then, 10 mL of ACN was added to the tube, which was vortexed again before adding an extraction salt packet. The mixture was vigorously shaken by hand, vortexed for 5 min and centrifuged at 7000 rpm for 5 min. During the optimisation process, to avoid high ion suppression in the final extracts due to the lack of a clean-up step, and to prevent the loss of the compounds by evaporation, 1 mL of the ACN phase was transferred to a 10 mL volumetric flask and taken up to the final volume with ultrapure water, instead of preconcentrating the extracts before the injection. The solvent volume and vortex time conditions for the extraction were set based on our previous experience [32]. It should be noted that, since the lipidic content of the samples can affect the efficiency of the extraction, two seafood species with different lipid percentages were chosen for the optimisation: hake (low lipid content) and mackerel (high lipid content).

**Figure 2:**
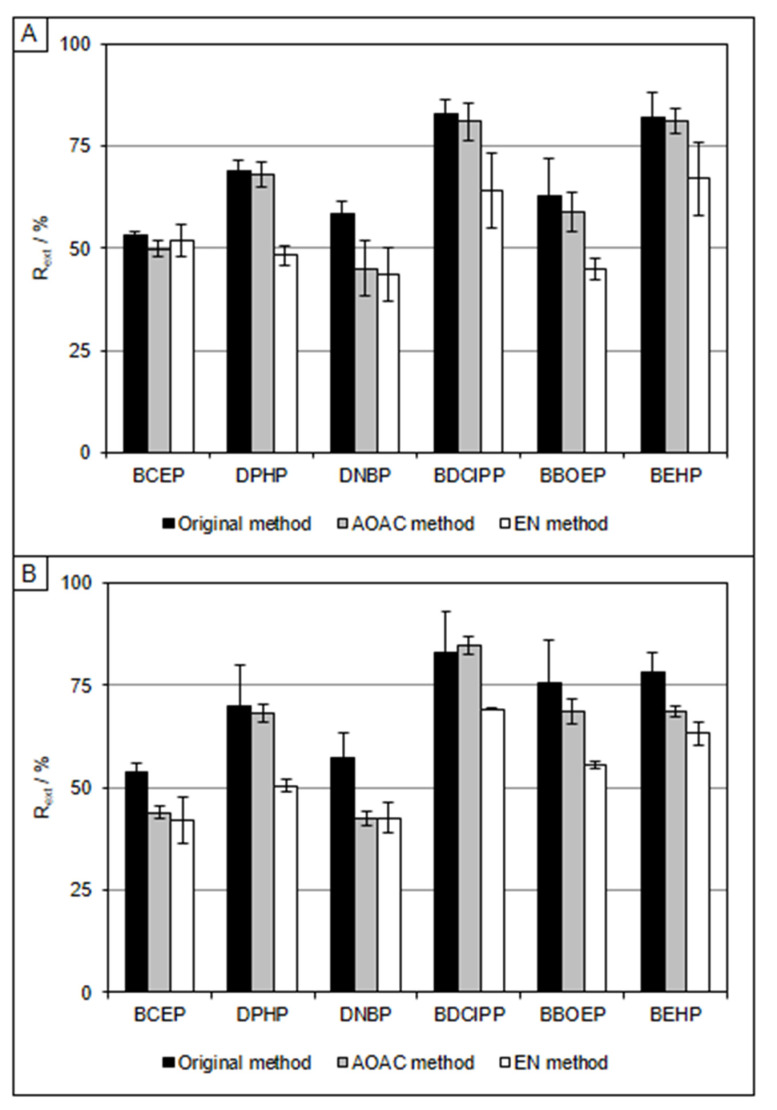
Extraction recoveries obtained for hake (**A**) and mackerel (**B**) samples using different extraction methods with acetone.

The R_ext_ obtained with all three extraction methods ranged from 10 to 65% for most compounds; therefore, the use of other extraction solvents was tested. Based on a PLE method for extracting several OP triesters and diesters from fish [26], we decided to switch the extracting solvent to acetone. Following the same procedure as described above and only changing the 10 mL of ACN for 10 mL of acetone, the R_ext_ of all compounds increased significantly (Figure 2). The EN extraction salts yielded the lowest R_ext_ for all compounds except BCEP in hake (42–69%). The original method yielded the highest R_ext_ values, ranging from 53 to 83% for both species; hence, we selected the original salt packets for the extraction.

We also evaluated the change of R_ext_ with two and three consecutive extraction cycles. R_ext_ increased more than 20% for most compounds when two extraction cycles were carried out. Three extraction cycles yielded slightly better R_ext_, but they also increased the ME by almost 20%. We therefore selected two consecutive extraction cycles for the present procedure, which yielded a R_ext_ of 60–79% for both hake and mackerel.

### 3.3. Clean-Up Strategies

Several clean-up strategies were tested to decrease the high ion suppression observed in the final extracts (up to −78%). To evaluate the performance of the different procedures, ME values were calculated by spiking samples after the clean-up step and comparing the signal of the analytes with the signal of a standard solution. We also evaluated the retention of the target compounds in the different sorbents used to clean the extracts by comparing the response of the compounds for an extract spiked after the clean-up step with the response obtained for an extract spiked before the clean-up step. All procedures were tested on the same two seafood species chosen for the QuEChERS optimisation.

For a quick and simple clean-up, we tested Lipifiltr^®^ push-thru purification cartridges, which are specifically designed to remove lipids from fatty QuEChERS extracts [32,33]. However, all the target compounds were completely retained in the cartridges, so this strategy was promptly dismissed. Then, we tried using dSPE since it is the most commonly used technique for the clean-up of QuEChERS extracts. PSA and C_18_ are some of the sorbents typically used to remove fatty acids and overall high lipid content from samples [34], so they were tested separately. To do so, 20 mL of acetone spiked with the analytes of interest was transferred to a tube containing 200 mg of sorbent. The tube was vortexed for 3 min and centrifuged at 7000 rpm for 5 min. The organic solvent was then evaporated to ~100 µL and reconstituted to 1 mL with ultrapure water before being injected into the LC-HRMS system. OP diesters were highly retained in PSA, but low retention (<11%) was obtained when C_18_ was used, making the latter an appropriate sorbent for dSPE. However, when we applied the procedure to sample extracts, we detected the formation of a surface lipid film during the evaporation step. This film hindered the evaporation of the organic solvent, making the process too time-consuming and evidencing the need to change the solvent before evaporation.

Taking into account the high retention of the target compounds in PSA, we proposed an SPE procedure with a lab-packed PSA cartridge to retain the analytes of interest and eliminate the interferences. First, we compared the retention of cartridges packed with different amounts of PSA (200 mg, 500 mg, 1 g and 2 g). The cartridges were conditioned with 5 mL of MeOH followed by 5 mL of acetone. Then, 20 mL of acetone spiked with the compounds of interest was loaded onto the cartridges and the analytes were eluted with 5 mL of 5% NH_4_OH in MeOH. The extracts were concentrated to ~100 µL and reconstituted to 1 mL with ultrapure water before the injection. The best results were obtained with 2 g PSA cartridges.

Next, we carried out the same procedure with sample extracts to evaluate the ME obtained after the clean-up step, but the retention of the analytes dropped from ~100% to less than 20% for both hake and mackerel. This was due to the saturation of the sorbent. Therefore, we reduced the quantity of sample extracted to 100 mg. Even though the results improved, the retention of the compounds was still low (<40%) so we tried to increase it by reducing the presence of organic solvent in the extracts. We evaporated the QuEChERS extract to ~5 mL and diluted it with 5, 10, 15 and 20 mL of ultrapure water. Overall, the best retention values for both the hake and mackerel samples were obtained when the extract was diluted with 10 mL of ultrapure water. The ME values obtained using any of the water volumes ranged from −2 to −70% for hake and from −1 to −51% for mackerel. We chose to dilute the extract with 10 mL of ultrapure water for the SPE clean-up.

Due to the high ME obtained for some analytes, two strategies were assessed to clean up the SPE extract further. First, we tested a washing step with different volumes of ultrapure water/acetone (2:1, *v*/*v*) after loading the sample onto the cartridges. While no significant differences in ME were observed, some compounds were eluted in this washing step, and therefore it was discarded. Second, lab-packed Florisil and C_18_ cartridges were connected to the top of the PSA cartridge to retain interfering substances [31,35]. In both cases, the ME was either similar or just slightly better, so this strategy was also rejected and no further modifications were made to the method.

There is little information in the literature on the optimisation of the clean-up steps for seafood samples or the matrix effects obtained. It was therefore not possible to compare our results with previous studies.

### 3.4. Method Validation

Hake and mackerel samples were used to evaluate the performance of the method with high- and low-lipid content seafood samples. To do so, we determined relative recoveries (R_rel_), method limits of detection (MLOD), method limits of quantification (MLOQ), repeatability and reproducibility. The validation results are summarised in Table 2.

We used d_8_-BCEP, d_10_-DPHP, d_18_-DNBP, d_10_-BDCIPP and d_8_-BBOEP as surrogate standards for validating the method and quantifying the samples to compensate for the ME and the low recoveries of some compounds. The surrogates for BCEP, DPHP, DNBP, BDCIPP and BBOEP were the corresponding deuterated analogues. Since no deuterated analogue was available for BEHP, calibration curves were calculated for that compound with each of the surrogate standards. The use of d_8_-BBOEP yielded the best linearity and R_rel_ values so it was selected as the surrogate for BEHP. Quantification was therefore carried out using instrumental calibration curves with surrogates.

**Figure 3:**
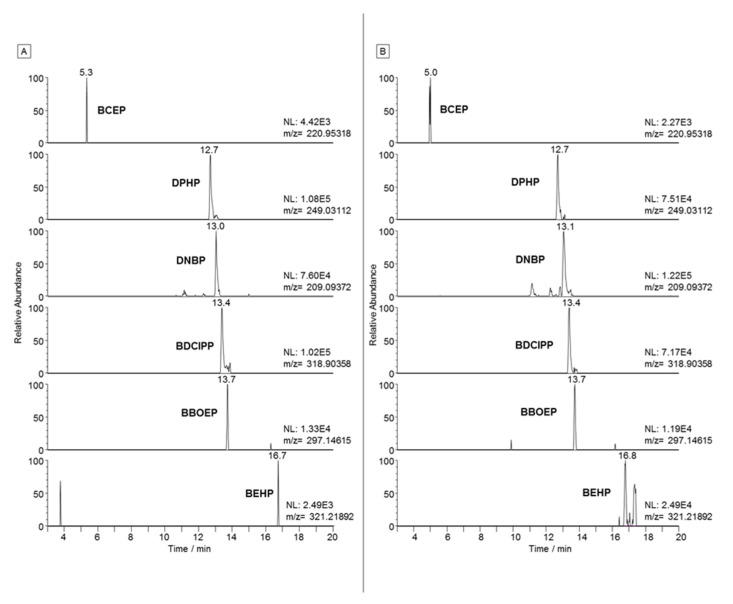
LC-HRMS extracted ion chromatograms of a hake (**A**) and mackerel (**B**) samples spiked at 100 ng/g (d.w.).

R_rel_ was calculated in triplicate for hake and mackerel samples spiked at 100 ng g^−1^ (d.w.) before the extraction and results are shown in Table 2. Figure 3 shows the chromatograms of the corresponding spiked samples. Then, the concentration obtained using the instrumental calibration curves with surrogates was compared with the spiked concentration. As expected, BEHP was the only compound for which R_rel_ was much lower than 100%. R_rel_ was therefore applied to the results for BEHP, depending on the lipid content of the sample.

MLOD and MLOQ values were evaluated by spiking the samples at several concentrations. The MLOD was established as the concentrations with a signal-to-noise ratio of the quantification ion equal to three. MLOQ was defined as the lowest concentrations in the calibration curve. As can be seen in Table 2, the values obtained were similar for both hake and mackerel samples. The MLOD values ranged from 1.0 to 7.5 ng g^−1^ (d.w.) for DPHP, DNBP and BDCIPP, and from 25 to 50 ng g^−1^ (d.w.) for BCEP, BBOEP and BEHP. The MLOQ values ranged from 2.5 to 10 ng g^−1^ (d.w.) for DPHP, DNBP and BDCIPP, and from 50 to 75 ng g^−1^ (d.w.) for BCEP, BBOEP and BEHP, in both representative species. The differences in the limits are due to lower R_ext_ values in the QuEChERS extraction and lower retention in the PSA cartridges for BCEP, BBOEP and BEHP.

Repeatability (intra-day) and reproducibility (day-to-day), expressed as relative standard deviation (RSD%, *n* = 5, 100 ng g^−1^ (d.w.)), were below 15 and 21%, respectively, for all compounds except for BEHP. For that compound, repeatability and reproducibility were both above 30%.

Since repeatability and reproducibility were very high for BEHP, and R_rel_ was very low, we can confirm that the surrogate chosen for quantification is inadequate. If BEHP is detected in any seafood samples the quantification should therefore be considered tentative or an estimation.

### 3.5. Application to Seafood Samples

**Figure 4:**
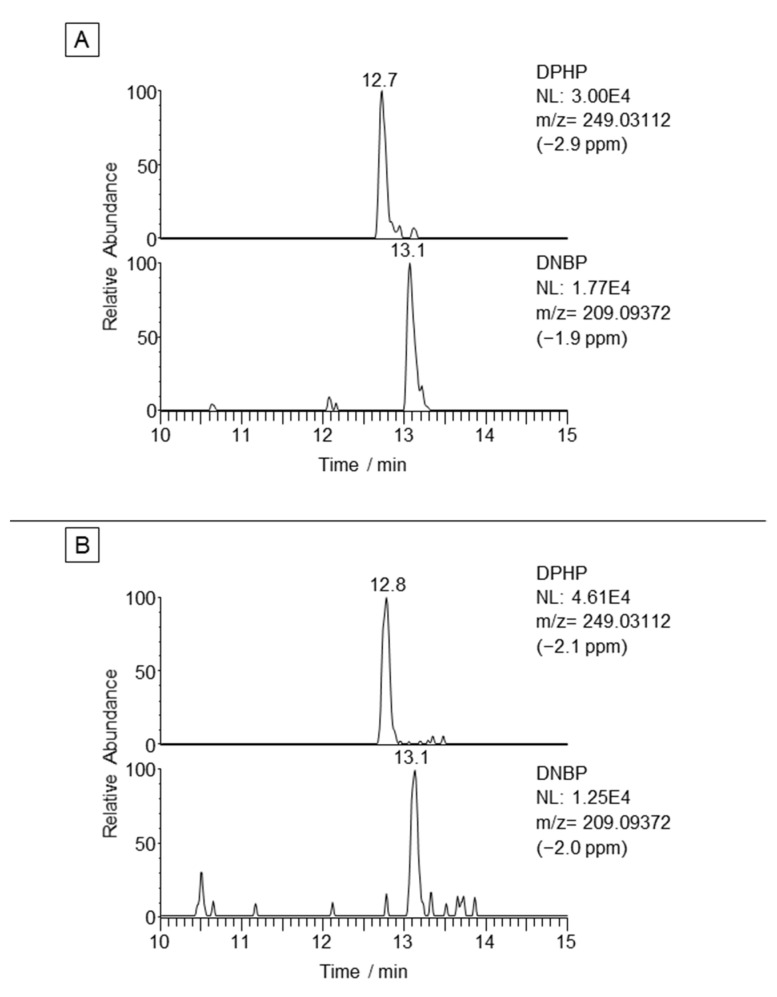
LC-HRMS extracted ion chromatogram of a hake (**A**) and a tuna (**B**) sample.

The optimised QuEChERS/SPE/LC-HRMS method was applied to samples of eight seafood species to determine OP diesters. Four species with low lipid content (hake, sole, cod and squid) and four species with a high lipid content (sardine, tuna, mackerel and salmon) were bought from local markets. We evaluated the presence of OP diesters in triplicate for each sample using the retention time, the exact mass of the analytes and the ratio between the quantifier and the qualifier ions to confirm the presence of the detected compounds. Figure 4 shows the LC-HRMS extracted ion chromatograms for the hake and tuna samples.

DPHP and DNBP were determined in all the samples and their presence was confirmed with a mass error below 5 ppm and the expected ion ratios. The concentrations of the compounds are shown in Table 3. The concentrations of DPHP ranged from 42 to 100 ng g^−1^ (d.w.). The concentrations of DNBP were similar and ranged from 30 to 79 ng g^−1^ (d.w.). No differences could be observed in the results between seafood with low or high lipid content, which is in line with previous reports [36]. There is little information on the occurrence of OP diesters in seafood samples, and the results are calculated depending on the lipid weight or the wet weight of the samples. This makes comparing our results with previous studies challenging. Nevertheless, the concentrations found in this study are within the range of those reported by Hou et al. [26], who found values ranging from 8 to 284 ng g^−1^ (l.w.) for DPHP and from 9 to 161 ng g^−1^ (l.w.) for DNBP. Zheng et al. [24], in contrast, found much lower concentrations than those found in this study (up to 2.35 ng g^−1^ (w.w.) for both compounds).

BCEP, BDCIPP, BBOEP and BEHP were not detected in any of the samples. Similar results were obtained by Zheng et al. [24], who found values ranging from non-detected to concentrations below 0.3 ng g^−1^ (w.w.). However, these results are not strange. Several studies demonstrate that when the tissue-specific distributions of both OP triesters and diesters are compared, muscle tissue usually contains these compounds at some of the lowest concentrations or even at undetectable levels. This could be because OP triesters seem to accumulate and metabolise in metabolically active tissues such as the liver and kidneys [25,26].

It should also be noted that a significant positive correlation has been confirmed between the concentration of DNBP and DPHP and the concentration of their parent compounds in several fish [24]. This makes them a useful tool for evaluating the exposure of fish to OP triesters.

## 4. Conclusions

In this study, we have developed a simple method for determining six OP diesters in seafood. The method consists of a QuEChERS extraction, followed by an SPE clean-up and LC-HRMS. Several clean-up strategies were tested and the best results were obtained by using PSA as the sorbent for SPE. The procedure was successfully validated for both low and high lipid content seafood, yielding good relative recoveries and providing satisfactory reproducibility and sensitivity. We then analysed eight widely consumed seafood species with different lipid contents using this method. DPHP and DNBP were detected and quantified in all eight samples, with concentrations ranging from 30 to 100 ng g^−1^ (d.w.). BCEP, BDCIPP, BBOEP and BEHP were not detected in any of the samples. There were no significant differences in the concentrations of OP diesters depending on the lipid content of the sample. The results obtained demonstrate that this method is suitable for determining OP diesters in seafood regardless of the lipid content of the samples.

## Figures and Tables

**Table 1:** Chemical structure of the analytes studied and exact masses of their quantifier and qualifier ions.

Compound and Structure	Quantifier Ion (*m/z*)	Qualifier Ions (*m/z*)
Bis(2-chloroethyl) phosphate (BCEP) 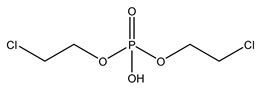	[C_4_H_8_PO_4_Cl_2_]^−^ 220.95318	[PO_3_]^−^ 78.95796
Diphenyl phosphate (DPHP) 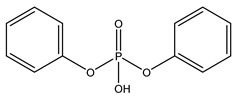	[C_12_H_10_PO_4_]^−^ 249.03112	[C_6_H_5_O]^−^ 93.03349 [C_6_H_4_PO_3_]^−^ 154.98926
Dibutyl phosphate (DNBP) 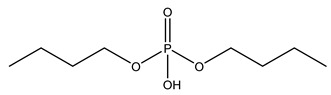	[C_8_H_18_PO_4_]^−^ 209.09372	[PO_3_]^−^ 78.95796 [C_4_H_10_PO_4_]^−^ 153.03112
Bis(1,3-dichloro-2-propyl) phosphate (BDCIPP) 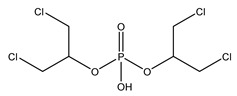	[C_6_H_11_PO_4_Cl_3_^37^Cl]^−^ 318.90358	[PO_3_]^−^ 78.95796
Bis(2-butoxyethyl) phosphate (BBOEP) 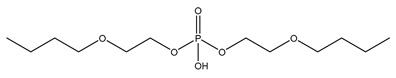	[C_12_H_26_PO_6_]^−^ 297.14615	[PO_3_]^−^ 78.95796 [C_6_H_14_PO_5_]^−^ 197.05734
Bis(2-ethylhexyl) phosphate (BEHP) 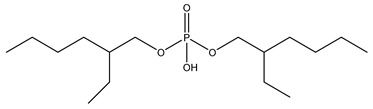	[C_16_H_34_PO_4_]^−^ 321.21892	[PO_3_]^−^ 78.95796 [C_8_H_18_PO_4_]^−^ 209.09372

**Table 2:** Retention time (t_R_), relative recovery (R_rel_), matrix effect (ME), method limit of detection (MLOD) and method limit of quantification (MLOQ) for two representative species.

Compound	t_R_ (min)	Hake (*Merluccius merluccius*)				Mackerel (*Scomber scombrus*)			
		R_rel_ (%)	ME (%)	MLOD (ng g^−1^)	MLOQ (ng g^−1^)	R_rel_ (%)	ME (%)	MLOD (ng g^−1^)	MLOQ (ng g^−1^)
BCEP	4.8	113	−2	25	50	107	−1	50	75
DPHP	12.6	108	−40	1.0	7.5	99	−45	1.0	2.5
DNBP	13.0	138	−39	2.5	5.0	122	−35	1.0	2.5
BDCIPP	13.3	104	−24	2.5	5.0	100	−11	7.5	10
BBOEP	13.6	108	−28	25	50	89	−21	25	50
BEHP	16.6	38	−70	50	75	22	−56	50	75

**Table 3:** Concentrations (ng g^−1^ (d.w.)) and relative standard deviation (RSD%, *n* = 3) of organophosphate diesters found in seafood species with different lipid content.

Compound	Seafood with Low Lipid Content (<10%)				Seafood with High Lipid Content (>10%)			
	Hake (*Merluccius merluccius*)	Sole (*Solea solea*)	Cod (*Gadus morhua)*	Squid (*Loligo vulgaris)*	Sardine (*Sardina pilchardus*)	Tuna (*Thunnus thynnus*)	Mackerel (*Scomber scombrus*)	Salmon (*Salmo salar*)
DPHP	59 (7)	54 (4)	68 (4)	48 (8)	42 (1)	75 (4)	100 (1)	55 (10)
DNBP	67 (2)	36 (13)	54 (0.3)	32 (3)	33 (7)	50 (9)	79 (1)	30 (2)

## Data Availability

The data presented in this study are available on request from the corresponding author.
